# Supplementary material for: NGS method for parallel processing of high quality, damaged or fragmented input material using target enrichment
Source: PLoS One. 2024 May 29;19(5):e0304411. doi: 10.1371/journal.pone.0304411 (PMC11135680; doi:10.1371/journal.pone.0304411)
Supplement: Attachment — Submitted filename: Response to reviewers.docx [file pone.0304411.s005.docx]

**Response to reviewers**

**1. Please ensure that your manuscript meets PLOS ONE's style requirements, including those for file naming.** The PLOS ONE style templates can be found at
<https://journals.plos.org/plosone/s/file?id=wjVg/PLOSOne_formatting_sample_main_body.pdf> and
<https://journals.plos.org/plosone/s/file?id=ba62/PLOSOne_formatting_sample_title_authors_affiliations.pdf>

**Author reply:** The manuscript has been edited according to the style requirements.

**2. Thank you for stating the following in the Competing Interests section:
 "VW has received reimbursement of travel costs and speaker’s honoraria from Illumina."**

Please confirm that this does not alter your adherence to all PLOS ONE policies on sharing data and materials, by including the following statement: "This does not alter our adherence to PLOS ONE policies on sharing data and materials.” (as detailed online in our guide for authors <http://journals.plos.org/plosone/s/competing-interests>). If there are restrictions on sharing of data and/or materials, please state these. Please note that we cannot proceed with consideration of your article until this information has been declared.

Please include your updated Competing Interests statement in your cover letter; we will change the online submission form on your behalf.

**Author reply:** The Competing Interests section has been clarified and added to the cover letter.

**3. In the online submission form, you indicated that "All relevant data are contained within the manuscript. Data of Figures 2-4 can be provided upon request."**

All PLOS journals now require all data underlying the findings described in their manuscript to be freely available to other researchers, either 1. In a public repository, 2. Within the manuscript itself, or 3. Uploaded as supplementary information.
This policy applies to all data except where public deposition would breach compliance with the protocol approved by your research ethics board. If your data cannot be made publicly available for ethical or legal reasons (e.g., public availability would compromise patient privacy), please explain your reasons on resubmission and your exemption request will be escalated for approval.

**Author reply:** The data has been added as supplementary data; S1 Table (Fig 2-3) and S2 Table (Fig 4).

**4. Your ethics statement should only appear in the Methods section of your manuscript.** If your ethics statement is written in any section besides the Methods, please move it to the Methods section and delete it from any other section. Please ensure that your ethics statement is included in your manuscript, as the ethics statement entered into the online submission form will not be published alongside your manuscript.

**Author reply:** The ethics statement has been moved to the Materials and Methods section.

**5. Please review your reference list to ensure that it is complete and correct.** If you have cited papers that have been retracted, please include the rationale for doing so in the manuscript text, or remove these references and replace them with relevant current references. Any changes to the reference list should be mentioned in the rebuttal letter that accompanies your revised manuscript. If you need to cite a retracted article, indicate the article’s retracted status in the References list and also include a citation and full reference for the retraction notice.

**Author reply:** The references have been modified according to the Vancouver style.

**Additional Editor Comments:**

When responding to reviewer comments, please make sure to clearly elaborate/explain differences in results from FFPE1-4 and Horizon reference samples. Also, differentiate your research article from a lab protocol, based on your provided data.

**Author reply:** This has been done accordingly.

**Comments to the Author**

**5. Review Comments to the Author**

Please use the space provided to explain your answers to the questions above. You may also include additional comments for the author, including concerns about dual publication, research ethics, or publication ethics. (Please upload your review as an attachment if it exceeds 20,000 characters)

**Reviewer #1:** Lyander et al. “NGS method for parallel processing of high quality, damaged or fragmented input
material using target enrichment”
In this article, the authors describe a procedure for NGS library preparation form different types of samples.
I have the following questions/comments:

**1. Were the same input amounts used for FFPE1-4 and the Horizon reference samples?**

**Author reply:** The input amounts used for the samples have been added to the Methods section, in “Enzymatic fragmentation of gDNA”.

**2. What QC metrics are used for the 2 panels described by the authors?** The reported duplication rates, target coverage and %target bases for the FFPE samples would, in many clinical laboratories, be considered as failures or not passing QC.

**Author reply:** This could be considered failed based on the clinical application used. To improve the QC metrics a larger amount of DNA from the FFPE material could be used. In addition, deeper sequencing can improve the QC metrics. The samples used in this case were poor quality DNA intended to show how data could be improved with an alternative laboratory protocol, and may not be fully representative for all clinical cases.

**3. Can the authors comment on the differences in QC metrics in Figure 3 and 4,** which are using different samples and panels. The results seem better with the Horizon reference samples and panel 2. Do the authors think this is due to the difference in panels/probes, or due to a difference between “real” samples and commercial reference samples?

**Author reply:** The results seem better with the Horizon reference samples compared to the FFPE samples. This is likely due to the difference in quality between the commercial formalin-comprised Horizon samples (not FFPE material) HD798, HD799 and HD803 with a DIN value of 7.3, 3.9 and 1.7, respectively, and the formalin fixed paraffin embedded patient samples with DIN 1.0, 1.1, 1.0, 1.7.

**4. Table 4.** Can the authors also provide the quality score for the variant calls?

**Author reply:** The quality scores for the variant calls have been added to supplementary tables 3 and 4.

**5. Table 4.** Can the authors include data for HD777 in the table.

**Author reply:** This has been added in table 5.

**6. Can the authors also include the total number of variants detected for the samples?** In many cases, detecting the expected variants is not the problem with compromised samples, but instead with high duplication rates, early PCR errors result in a high number of artifactual calls and a large number of false positive variants.

**Author reply:** The total numbers of variants detected have been added to supplementary tables 3 and 4.

**Reviewer #2:** The manuscript entitled "NGS method for parallel processing of high quality, damaged or fragmented input
material using target enrichment" described a joint procedure for preparing enriched
DNA libraries from high molecular weight DNA and DNA from formalin-fixed, paraffinembedded tissue, fresh frozen tissue material, as well as cell-free DNA.

- The Authors should provide the expand forms for all acronyms, including gene acronyms, through the text when they first appear.
- Gene acronyms should be written in italics.

**Author reply:** This has been changed accordingly.

**Reviewer #3:** The sutdy was conducted in a very relevant topic. The author very elaborately detailed an alterantive in a challenging issuee. I onlye suggest if author provide a pictorial diagram outlining the steps for the workflow, highlighting important QC steps which one should be aware of, will be helpful for the readers.

**Author reply:** Criteria of ≥ 187.5 ng (under Quant-iT) has been added to figure 1.
